# *Crocodylus acutus* (American crocodile) bite marks on a nest data logger

**DOI:** 10.7717/peerj.8577

**Published:** 2020-02-17

**Authors:** Stephanie K. Drumheller, Jennifer H. Nestler, Caitlin E. Hackett Farris, Seth C. Farris, Frank J. Mazzotti

**Affiliations:** 1 Department of Earth and Planetary Sciences, University of Tennessee, Knoxville, TN, United States of America; 2 Department of Wildlife Ecology and Conservation, Fort Lauderdale Research and Education Center, University of Florida, Fort Lauderdale, FL, United States of America

**Keywords:** Crocodylia, Parental care, Bite force, Tooth mark, Nest monitoring

## Abstract

Several data loggers deployed to monitor temperature and humidity of *Crocodylus acutus* (American crocodile) nests in South Florida could not be located after hatching. One badly damaged data logger was retrieved, providing insight into the possible fate of the others. Using a taphonomic approach, we identified numerous indentations, consistent with crocodylian bite marks, and inconsistent with potential mammalian or squamate bites. It seems most likely that the data logger was damaged by the nesting *C. acutus* rather than during attempted nest predation. Estimated bite forces for reproductive age, female *C. acutus* exceed the predicted material properties of the data logger’s housing, suggesting that the bites were exploratory in nature. We suggest that data loggers be removed prior to hatching or permit remote data storage.

## Introduction

The American crocodile (*Crocodylus acutus*) has a broad distribution throughout North America, South America, and the Caribbean basin. In the United States, it is limited to the southern portion of the state of Florida, where it is typically found in coastal and estuarine habitats. It is a federally threatened species that may be affected by ecosystem restoration projects, particularly those related to the greater Everglades and Florida Bay (Mazzotti et al., 2007a; Mazzotti et al., 2009). As documented across other crocodylian species (Thorbjarnarson, 1996; Cañas & Anderson, 2002; Piña, Merchant & Verdade, 2015), environmental changes within American crocodile nests can significantly affect the health and survival of the developing young (Lutz & Dunbar-Cooper, 1984; Mazzotti et al., 1986; Mazzotti, Kushlan & Dunbar-Cooper, 1988; Mazzotti, 1989; Charruau, 2012; Charruau, Hénaut & Álvarez Legorreta, 2013; Murray et al., 2016). *Crocodylus acutus* exhibits temperature-dependent sex determination (Murray et al., 2016; Charruau, Cantón & De la Cruz, 2017), and developing embryos are vulnerable to both flooding and desiccation (Mazzotti, Kushlan & Dunbar-Cooper, 1988; Kushlan & Mazzotti, 1989; Charruau, Thorbjarnarson & Hénaut, 2010). Therefore, monitoring microenvironmental conditions in the nest, especially temperature and moisture, is a critical component of conservation efforts focused on this species.

In Florida, *C. acutus* populations have been expanding thanks to targeted conservation efforts (Kushlan, 1988; Mazzotti, 1989; Mazzotti et al., 2007b; Cherkiss, Romañach & Mazzotti, 2011), leading to the species being reclassified from endangered to threatened in 2007 (Federal Register 72:13027-13047). As part of ongoing monitoring of *C. acutus*, we placed data loggers in active nesting sites in 2017. The devices were buried in known nests and retrieval was attempted within two days of hatching. Despite logging nest locations with Global Positioning System (GPS) coordinates and physically flagging the devices, several data loggers used in this study could not be found. However, one heavily damaged data logger was discovered in close proximity to a monitored nest, providing insights into one potential fate of the missing devices. Here we describe the monitoring process and damage to the data logger, use taphonomic methods to evaluate the origin of the damage, and provide suggestions to mitigate this type of data loss.

## Materials & Methods

**Figure 1:**
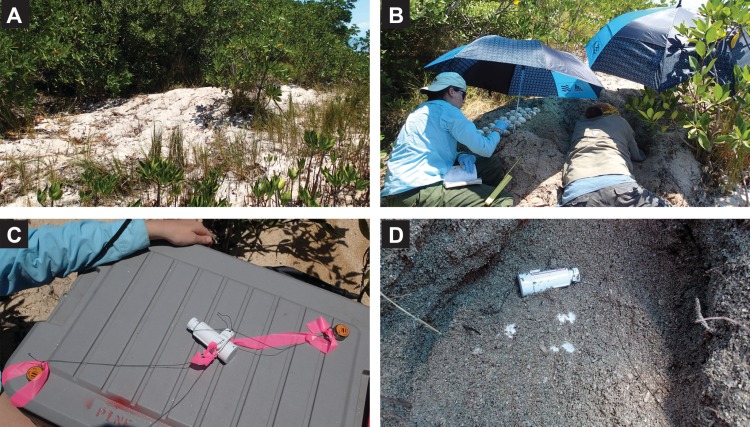
*Crocodylus acutus* nest excavation and data logger placement. (A) *Crocodylus acutus* nest, prior to excavation and data logger placement. (B) Nest excavation and egg removal. (C) Example of data loggers, tied together and flagged for recovery. (D) Data logger placement during egg reburial.

For the 2017 *C. acutus* breeding season, we selected six nesting sites for data logger placement, all located within southern coastal Florida. The University of Florida approved the research (IACUC #201809072) and the US Fish and Wildlife Service (TE077258-2) and the National Park Service (EVER-2016-SCI-0027) provided permits to perform this pilot study. Prior to excavation, nests were photographed (Fig. 1). Nests were then carefully excavated by researchers and measured, largely following methods outlined by Charruau (2012).

Once all eggs were safely removed and the egg chamber measured, data logger placement occurred. We used Onset TidbiT v2 (UTBI-001) to record temperature and Onset HOBO Pro v2 Temp/RH (U23-001) to record relative humidity and temperature. One temperature logger, attached to the other two data loggers by 150 lb (68 kg) test microfilament braided line, was placed at the bottom of the nest cavity. After returning half of the eggs to the nest, a relative humidity/temperature logger was placed in the middle of the clutch. Finally, a second temperature logger was placed at the top of the clutch after the rest of the eggs had been carefully returned. A buried microfilament braided line joined the middle logger to the base of a nearby tree to anchor the data loggers in place during nest excavation by the female crocodile. A piece of pink tape was tied to each group of loggers to help make them more visible during recovery. The nest was then completely covered and returned to the state in which it had been found.

We recorded GPS coordinates of the nest before removing all other equipment from the site. Nests were periodically observed throughout the incubation period, but were otherwise undisturbed by researchers. We attempted to recover data loggers within two days of hatching and nest excavation by the female crocodiles, but of the 18 devices that were deployed, 7 were never found.

**Figure 2:**
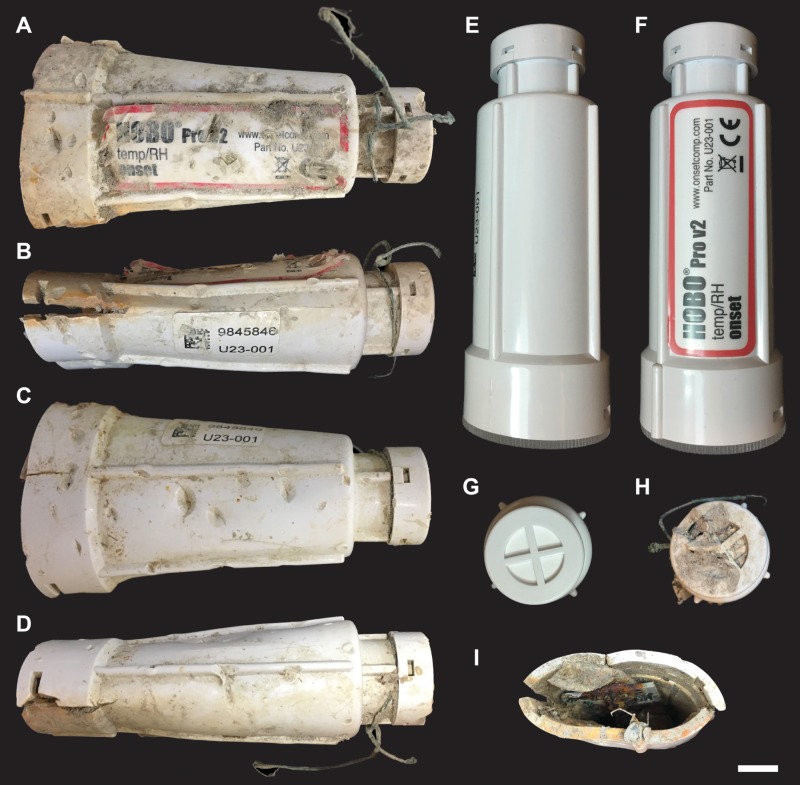
Damaged and pristine Onset HOBO Pro v2 Temp/RH (U23-001) data loggers. (A–D) Damaged data logger from four angles, representing the full circumference of the device. Bite marks and evidence of compression and cracking are present in all four views. (E–G) Pristine data logger in side and top views. (H–I) Damaged data logger in top and bottom views, showing the degree of compression and cracking in the damaged device. Scale bar = one cm.

On September 4, 2018, field crews retrieved a damaged Onset HOBO Pro v2 Temp/RH (U23-001) from the water near the nest, about a month and a half after hatching. The data logger exhibited obvious crushing and impact damage (Fig. 2). Unfortunately, no data could be retrieved, because the damage had exposed the internal sensors and data storage components to saltwater and sediment.

The patterns of impact damage seemed to be congruent with bite marks. Predation is known to be a significant cause of egg mortality in crocodylians (e.g., Somaweera, Webb & Shine, 2011; Campos et al., 2016), including *C. acutus* (e.g., Kushlan & Mazzotti, 1989; Mazzotti, 1989). Therefore, the identity of the trace maker was of special interest. The polyvinyl chloride (PVC) housing for the unit does not exhibit identical material properties to bone, but the generalized structure of the data logger (an elongate tube comprising a hard, dense exterior and a hollow interior) is suitably analogous to allow rough comparisons. Therefore, we are able to turn to the taphonomic literature, specifically regarding bone surface modifications, to identify the cause of the damage to the recovered data logger.

Tooth crown size and shape are reflected in the indented surface during biting events, and actualistic taphonomic research on these bone surface modifications often focuses on methods to differentiate traces left by different groups of trace makers (Binford, 1981; Fisher, 1995). To determine which organism made the bite marks, we measured length (long axis) and width (perpendicular to the long axis) of each identifiable tooth mark using digital calipers. Bite mark types, as defined by Binford’s (1981) bite mark identification and classification scheme, were recorded. This nomenclature divides bite marks by depth of trace penetration and presence or absence of lateral movement of the associated tooth. In general, pits and scores indent, but do not fully pierce the bitten substrate while punctures and furrows do. Additionally, scores and furrows are elongate structures associated with lateral tooth movement while pits and punctures are formed when the impacting forces only move perpendicular to the bitten substrate. We also noted any potentially diagnostic features of the traces (Table S1), especially bisected marks, which are associated with the prominent carinae of freshly erupted crocodylian teeth (Njau & Blumenschine, 2006; Drumheller & Brochu, 2014; Drumheller & Brochu, 2016; Njau & Gilbert, 2016), and edge marks, which are made when the side of a tooth, especially a prominent carina, contacts a prominent ridge on the bitten substrate (sensu D’Amore & Blumenschine, 2009). Finally, we consulted the material properties reports of the data logger components (UL Prospector, 2019) and compared these amounts with estimates of crocodylian bite forces (Erickson et al., 2012).

## Results

The PVC housing of the undamaged data logger was 10 cm long and stepped down from 3.5 cm to 2.25 cm in diameter (Fig. 2). It was white in color, with external labels recording brand, model, etc. Devices for measuring and recording temperature and relative humidity and transmitting the measured data were housed inside of the PVC casing, with caps sealing each end. Four structural ribs ran the length of the data logger, spaced equidistantly from one another.

The damaged data logger exhibited crushing damage and deformation. At its widest diameter, the housing had been compressed to roughly 5.2 cm by 2.4 cm and exhibited cracking perpendicular to both the long and short axes of the cross-sectional shape. The exterior of the housing was further damaged with indentations and associated impact damage.

Following Binford’s (1981) criteria for identifying different types of bone surface modifications, the indentations covering the data logger’s housing were consistent with bite marks, as they exhibit crushing and fracturing associated with repeated impact and compressive damage by multiple hard, dense structures, i.e., teeth. Again following Binford’s (1981) bite mark classification scheme, 85 pits and 12 scores were identified on the data logger. Furthermore, six edge marks (sensu D’Amore & Blumenschine, 2009) were present on some of the raised, structural ribs on the device’s housing. These types of bite marks often occur on raised crests or other prominent ridges on the bitten material and are caused when the sides of a tooth, rather than the apex, contacts bone. Measurements of these surficial modifications are provided in Table S1.

The pits ranged in size from 0.9 by 0.73 mm to 8.72 by 5.29 mm, making the largest of the traces inconsistent with any of the small-bodied, mammals, such as *Procyon lotor* and *Dasypus novemcinctus*, that have been documented feeding on crocodylian nests (Mazzotti, 1989; Somaweera, Webb & Shine, 2011; Campos et al., 2016). All observed pits were subround to fusiform in shape, indicative of conical, relatively homodont dentition. These tooth marks were therefore inconsistent with traces associated with the heterodont dentition of larger mammalian carnivores known from South Florida, including several species of *Canis* (Binford, 1981; Haynes, 1982; Haynes, 1983; Haglund, Reay & Swindler, 1988; Haglund, 1997), *Ursus americanus* (Haynes, 1982; Murad & Boddy, 1987; Murad, 1997; Saladié et al., 2011) *Lynx rufus*, and *Puma concolor* (Haynes, 1983; Labisky & Boulay, 1998; Domínguez-Rodrigo, 1999). In general, mammalian feeding behavior results in more morphologically complex scores and pits, caused by multicusped molars, and more concentrated damage on the ends of elongate structures, caused by gnawing behavior. Among potential non-mammalian predators, *Salvator merianae* and other large, invasive lizards, are present in the Everglades (e.g., Klug et al., 2015; Ketterlin Eckles et al., 2016). While these animals are known to predate crocodylian nests (Campos et al., 2016), again, their teeth are too small and the varanids in particular exhibit ziphodont dentition. These serrated teeth leave striated tooth marks (D’Amore & Blumenschine, 2009), a pattern that was inconsistent with the observed marks.

Fortunately, the bisected, fusiform shape of some of the identified pits suggests a probable trace maker. Modern crocodylian teeth are conical in shape with prominent carinae. With relatively freshly erupted, unworn crocodylian teeth, this morphology is expressed as a sub-score, or bisection, within the larger bite mark structure, a feature which is diagnostic of crocodylian trace makers (Njau & Blumenschine, 2006; Drumheller & Brochu, 2014; Drumheller & Brochu, 2016). A single hook score, often associated with an inertial feeding strategy, was also identified (Njau & Blumenschine, 2006; D’Amore & Blumenschine, 2009; Drumheller & Brochu, 2014; Drumheller & Brochu, 2016). Crocodylians also have the highest recorded bite force of any living animal (Erickson, Lappin & Vliet, 2003; Erickson et al., 2004; Erickson et al., 2014), further explaining the extensive crushing and deformation of the affected data logger. The most likely trace maker, given crocodylian nesting and hatching behavior, was the female *C. acutus* attending this specific nest.

To further explore the force required to damage the data logger, crocodylian bite force was calculated and compared to the material properties of the PVC used to form the device’s housing. There is a linear relationship between crocodylian mass and bite force across all modern species (Erickson et al., 2012). The formula generated by Erickson and colleagues was *y* = 1. 7113*x* + 335.72 in which *y* = taxon representative caniniform pressure in megapascals and *x* = mean body mass in kilograms. Nesting *C. acutus* were not captured as a part of this study, so exact metrics of the nesting female are not available. However, previous surveys of Florida *C. acutus* document that reproductive age females range from 2 to 3.5 m in length and 22 to 170 kilograms in mass (Mazzotti, 1983; Kushlan & Mazzotti, 1989). This yields predicted maximum bite pressures of 373.37 to 626.64 MPa. The reported Ball Indentation Hardness for the PVC housing of this data logger is only 65.02 to 104.80 MPa (UL Prospector, 2019), suggesting that the observed damage to the unit is more than within the capabilities of a nesting *C. acutus*.

## Discussion

After laying eggs and building a nest, some *Crocodylus acutus* females will stay in close proximity during incubation, though levels of nest attendance and defensive aggression have been documented to vary between individuals and populations (Dugan et al., 1981; Thorbjarnarson, 1986; Platt & Thorbjarnarson, 2000; Charruau & Hénaut, 2012). At the end of the incubation period, hatchlings produce vocalizations that signal the attending female to excavate the nest and aid in the hatching process (Vergne & Mathevon, 2008; Charruau & Hénaut, 2012). Once the eggs are uncovered, the female crocodile will then transport the hatchlings and hatching eggs to the water (Lang, 1987; Kushlan & Mazzotti, 1989; Charruau & Hénaut, 2012). It was almost certainly during this process that the female crocodile encountered the data logger described in this study.

Without direct observation of the behavior associated with the excavation and discovery of the data logger, it is difficult to speculate why the crocodile destroyed the retrieved data logger (and potentially the other two, which were never recovered from this nest). It is possible that the nesting female recognized the data loggers as something that did not belong in the nest and sought to remove them. Alternatively, the data loggers are somewhat similar to crocodile eggs in size and coloration, 42–54 mm × 70–80 mm (Thorbjarnarson, 1989), and so the device might have been picked up during the excavation and transportation process. The condition of the data logger does provide some insight into what followed.

The damage present on the device is not consistent with a feeding attempt and instead may have been the result of exploratory bites. Crocodylians exhibit an inertial feeding strategy, in which prey items are repositioned in the mouth by reversals of the jaw apparatus, brief release of the accelerated food item, and then rapid forward-moving closure of the jaws. This method of prey processing results in hook scores (sensu Njau & Blumenschine, 2006), in which the trajectory of a score changes direction abruptly on the bitten surface, and high densities of bite marks on the modified substrate, in which hundreds of individual tooth marks can be produced on a single element (Njau & Blumenschine, 2006; Drumheller & Brochu, 2014). Once positioned near the back of the mouth, crocodylians can then apply increasing pressure to break apart prey items before swallowing them (Cleurens & DeVree, 2000). Pressure was certainly applied to the damaged data logger, as indicated by the cracking and deformation of the PVC housing. However, even the minimum predicted bite force of a nesting female crocodile is much greater than the mechanical ability of the housing to withstand such an event. Additionally, more powerful bites are applied to reduce a prey item into manageable sizes in preparation for swallowing, something that would not have been required if the crocodile had intended to actually eat the small data logger. During exploratory bites, the application of comparatively minimal bite forces makes sense since identification rather than consumption was the end goal.

## Conclusions

Previous studies have successfully deployed similar data loggers with built in sensors and data storage (López-Luna et al., 2015; González-Desales et al., 2016; Murray et al., 2016). However, these projects did not experience the same apparent level of instrument loss (Charruau, 2012). In this study, we elected to leave the data loggers in place through hatching. While this decision minimized human interruption to the hatching process, it did expose the data loggers to excavation by the adult crocodiles. This resulted in higher rates of data loss.

If the requirements of a research project necessitate leaving data loggers in place through hatching, probe-style sensors connected by leads to external data storage devices could minimize data loss as could devices which transmit data periodically to a storage device. Alternatively, studies seeking to deploy data loggers for nest monitoring could either remove the devices prior to hatching or explore methods for reducing data loss. In this case, changes to these self-contained systems might warrant exploration. Considering their potential for extremely high bite forces, attempting to create a housing that can withstand crocodylian attack seems unlikely. However, changes to data logger appearance (i.e., not roughly crocodile egg-sized and -shaped) might prompt a change in crocodylian response to the devices, though whether these changes would prove to positive or negative remains to be seen. If self-contained data loggers must be used and must be left in place through hatching given the parameters of future nest monitoring projects, similarly high rates of device loss may be expected, given crocodylian nesting behavior.

##  Supplemental Information

10.7717/peerj.8577/supp-1 Table S1 Bite mark types and dimensions Classifications and measurements of bite marks on damaged data logger.

## Additional Information and Declarations

### Field Study Permissions

The following information was supplied relating to field study approvals (i.e., approving body and any reference numbers): Permits were obtained from the U.S. Fish and Wildlife Service (TE077258-2) and the National Park Service (EVER-2016-SCI-0027).

### Data Availability

The following information was supplied regarding data availability: The raw data is available in the Supplementary Files.
