# Supplementary material for: Crocodylus acutus (American crocodile) bite marks on a nest data logger
Source: PeerJ. 2020 Feb 17;8:e8577. doi: 10.7717/peerj.8577 (PMC7032054; doi:10.7717/peerj.8577)
Supplement: Table S1 — Classifications and measurements of bite marks on damaged data logger. [file peerj-08-8577-s001.docx]

| Mark  Number | Long Axis (mm) | Short Axis (mm) | Mark  Type | Notes |
| --- | --- | --- | --- | --- |
| 1 | 6.82 | 4.18 | Pit |  |
| 2 | 3.24 | 2.25 | Pit |  |
| 3 | 3.62 | 2.57 | Pit |  |
| 4 | 2.42 | 2.08 | Pit |  |
| 5 | 4.4 | 1.84 | Pit |  |
| 6 | 3.25 | 2.32 | Pit |  |
| 7 | 8.72 | 5.29 | Pit |  |
| 8 | 4.94 | 4.34 | Pit |  |
| 9 | 6.66 | 6.04 | Pit |  |
| 10 | 6.54 | 3.72 | Pit |  |
| 11 | 2.77 | 1.66 | Pit |  |
| 12 | 5.26 | 5.21 | Pit |  |
| 13 | 4.35 | 2.22 | Pit |  |
| 14 | 6.09 | 0.68 | Score | Intersects marks 12, 13 |
| 15 | 2.91 | 2.81 | Pit |  |
| 16 | 3.62 | 1.91 | Pit | Intersects mark 17 |
| 17 | 2.29 | 1.46 | Pit | Intersects mark 16 |
| 18 | 3.65 | 2.96 | Pit |  |
| 19 | 6.23 | 4.32 | Pit |  |
| 20 | 1.53 | 1.05 | Pit |  |
| 21 | 2.15 | 1.42 | Pit |  |
| 22 | 2 | 0.18 | Score |  |
| 23 | 5.06 | 4.38 | Pit |  |
| 24 | 3.71 | 2.41 | Pit | Associated edge mark |
| 25 | 4.84 | 2.55 | Pit |  |
| 26 | 4.96 | 2.6 | Pit |  |
| 27 | 3.05 | 1.91 | Pit |  |
| 28 | 4.14 | 4.95 | Pit |  |
| 29 | 3.71 | 2.13 | Pit |  |
| 30 | 2.78 | 1.59 | Pit |  |
| 31 | 4.39 | 2.14 | Pit |  |
| 32 | 1.99 | 1.02 | Pit |  |
| 33 | 2.66 | 1.4 | Pit |  |
| 34 | 2.35 | 0.59 | Pit |  |
| 35 | 1.39 | 0.28 | Pit |  |
| 36 | 3.21 | 0.47 | Pit |  |
| 37 | 4.17 | 2.28 | Pit |  |
| 38 | 1.61 | 1.24 | Pit |  |
| 39 | 2.37 | 1.42 | Pit |  |
| 40 | 8 | 4.41 | Pit |  |
| 41 | 6.14 | 1.81 | Score |  |
| 42 | 4.55 | 2.74 | Pit |  |
| 43 | 6.74 | 1.19 | Pit |  |
| 44 | 4.49 | 2.77 | Pit |  |
| 45 | 4.03 | 2.95 | Pit |  |
| 46 | 5.54 | 3.95 | Pit |  |
| 47 | 2.97 | 2.38 | Pit |  |
| 48 | 4.16 | 1.89 | Pit |  |
| 49 | 3.91 | 1.84 | Pit |  |
| 50 | 1.6 | 1.29 | Pit |  |
| 51 | 2.24 | 1.42 | Pit |  |
| 52 | 4.45 | 2.23 | Pit | Associated edge mark |
| 53 | 6.37 | 0.43 | Score |  |
| 54 | 4.32 | 2.84 | Pit |  |
| 55 | 2.64 | 1.74 | Pit | Associated edge mark |
| 56 | 4.43 | 3.43 | Pit | Intersects mark 57 |
| 57 | 3.94 | 3.4 | Pit | Intersects mark 56 |
| 58 | 3.54 | 1.18 | Pit |  |
| 59 | 4.35 | 2.11 | Pit |  |
| 60 | 7.68 | 4.78 | Pit |  |
| 61 | 5.93 | 3.95 | Pit |  |
| 62 | 3.72 | 2.64 | Pit | Associated edge mark |
| 63 | 5.42 | 3.86 | Pit | Associated edge mark |
| 64 | 2.77 | 1.9 | Pit |  |
| 65 | 2.62 | 1.91 | Pit |  |
| 66 | 17.92 | 0.32 | Score |  |
| 67 | 1.5 | 0.59 | Pit |  |
| 68 | 8.41 | 0.21 | Score | Hook score |
| 69 | 1.87 | 0.38 | Score | Hook score |
| 70 | 2.59 | 2.51 | Pit |  |
| 71 | 1.75 | 1.28 | Pit |  |
| 72 | 1.66 | 0.31 | Score |  |
| 73 | 9.36 | 0.3 | Score |  |
| 74 | 2.37 | 1.02 | Pit |  |
| 75 | 1.81 | 0.55 | Pit |  |
| 76 | 2.17 | 1.4 | Pit |  |
| 77 | 2.18 | 1.18 | Pit |  |
| 78 | 1.4 | 0.66 | Pit |  |
| 79 | 2.98 | 2.21 | Pit |  |
| 80 | 1.76 | 0.58 | Pit |  |
| 81 | 7.9 | 1.11 | Pit |  |
| 82 | 2.95 | 0.53 | Pit |  |
| 83 | 1.69 | 0.66 | Pit |  |
| 84 | 2.3 | 1.22 | Pit |  |
| 85 | 2.33 | 1.31 | Pit |  |
| 86 | 1.51 | 0.9 | Pit |  |
| 87 | 2.33 | 1.18 | Edge mark |  |
| 88 | 2.24 | 0.67 | Pit |  |
| 89 | 1.95 | 1.68 | Pit |  |
| 90 | 4.1 | 0.54 | Score |  |
| 91 | 3.6 | 0.43 | Score |  |
| 92 | 0.9 | 0.73 | Pit |  |
| 93 | 1.36 | 1.11 | Pit |  |
| 94 | 1.37 | 0.51 | Pit |  |
| 95 | 1.61 | 1.27 | Pit |  |
| 95 | 6.17 | 2.31 | Pit |  |
| 96 | 7.65 | 0.28 | Score |  |
| 97 | 5.38 | 1.96 | Pit | Associated edge mark |
